# Unveiling the Uniqueness of Crystal Structure and Crystalline Phase Behavior of Anhydrous Octyl *β*-D-Glucoside Using Aligned Assembly on a Surface

**DOI:** 10.3390/polym12030671

**Published:** 2020-03-17

**Authors:** Shigesaburo Ogawa, Isao Takahashi

**Affiliations:** 1 Department of Materials and Life Science, Faculty of Science and Technology, Seikei University, Tokyo 180-8633, Japan; 2 Department of Physics, School of Science and Technology, Kwansei Gakuin University, Sanda, Hyogo 669–1337, Japan

**Keywords:** octyl *β*-D-glucoside, crystal structure, phase transition, GI-WAXD, superlattice

## Abstract

Although the anomalous low crystallinity of octyl *β*-D-glucoside (*β*-OGlu) was first proposed more than 30 years ago, many fundamental aspects of its crystal structure and of the crystalline phase behavior of the pure substance have remained uncertain. In this paper, we employ grazing-incidence wide-angle X-ray-diffraction measurements using a two-dimensional detector (2D-GI-WAXD) and perpendicularly aligned crystalline films to demonstrate that *β*-OGlu forms crystal structures consisting of an intermediate phase—like a ripple phase with two large crystal-lattice constants, *a* and *c*, comparable to the lengths of its bilayer structures. Furthermore, solid-to-solid phase transitions accompanied by latent heat confirm the existence of a solid-solution-like phase consisting of a crystalline and a liquid-crystal (LC) phase, which persists over a 20 °C temperature range, in a single-component system. In addition, the system forms a superlattice, accompanied by a change in packing of the component sugars in the partial-melting state; this shift is different from the gel-crystal transition observed for a typical lipid system. These facts indicate that even in the crystalline phase formed from a single component, each individual *β*-OGlu molecule in a single-component phase plays a versatile role in the crystallisation and melting processes. These findings must somewhat explain the specific co-assembling features with proteins of *β*-OGlu, which has long been used empirically in biochemistry.

## 1. Introduction

As a pivotal lipid among numerous synthetic glycolipids, octyl *β*-D-glucoside (*β*-OGlu) has long attracted attention owing to its versatile biochemical applications. Particularly intriguing features are that the compound mimics membrane lipids, binding with protein molecules via hydrogen bonding or hydrophobic interactions, and it co-crystallizes with protein molecules [1,2,3,4,5,6,7,8,9]. In addition, it is intrinsically self-assembling, a characteristic that emerges from the various phase behaviors that depend upon the concentration and temperature. Owing to their great importance, both the lyotropic liquid-crystal (LC) and the thermotropic LC phase behaviors of *β*-OGlu, together with the surface activity and the effect of *β*-OGlu on other detergents or lipid self-assembling systems, has been extensively investigated during the past few decades by numerous researchers [2,10,11,12,13,14,15,16,17,18,19,20,21,22,23,24,25,26,27,28,29,30,31,32,33]. Immiscible solutions consisting of high- and low-concentration solutions of *β*-OGlu have been exploited for membrane protein purification [2]. The glass-transition behavior both in the absence and presence of ice, and the water-cluster-like responsive behavior under magnetic and electric fields have been demonstrated for the LC phases [23,25,26,28,29,30]. These physical properties of *β*-OGlu self-assembly must be directly or indirectly related to the specific features of individual *β*-OGlu interactions with proteins—and/or surrounding the proteins with self-assembling membrane domains—and they may further advance its use. However, despite the great advances in studies of the phases of *β*-OGlu, several significant fundamental aspects have unfortunately remained unconfirmed. In particular, the crystal phase formed by the *β*-OGlu molecules themselves under anhydrous conditions has remained uncertain.

The principal lines of evidence concerning the arrangement of glycolipids containing cyclic sugars have been based on investigations of a certain series of glycolipids that can be studied using single-crystal structural analysis [10,12,34,35]. These glycolipids typically form lamellar structures, with a highly parallel, packed, bilayer arrangement consisting of adjacent double layers. Thus, the lattice constants of typical cyclic sugar-based glycolipid crystals have been assumed to obey the following conditions: *a* >> *c* > *b* [10,12,34,35], with the unit cells each having one long and two short axes.

In contrast, a problematic issue for the study of the crystal phase of *β*-OGlu has been the lack of sufficiently large crystals for X-ray crystallographic structural analysis [11]. It is probable that the lack of such analyzes has greatly impeded further investigations of this substance. Instead, the crystal structures of pure octyl-*α*-D-glucoside (*α*-OGlu), an anomer compound of *β*-OGlu, and the mixed crystal consisting of one-by-one *α* and *β* anomers have been solved [15], but analyzes of pure *β*-OGlu crystals have been avoided. However, Dorset and Rosenbusch deduced a great difference in the packing structures of these crystals from the large difference in their melting enthalpies [11]. Therefore, the empirical assumption that the *β*-OGlu crystal structure can be modeled using the crystal structures of *α*-OGlu and of the mixture cannot be justified. Rather, it is worth recalling the uniqueness of the *β*-OGlu crystal structure as an ‘exclusive example’ from among the vast repertoire of previously studied glycolipid compounds. Indeed, compelling scientific and technological needs demand the determination of information about the unknown crystal phase in order to continue and develop further the science and technology involving this glucoside.

In addition, the phase assigned to crystalline *β*-OGlu, together with an unconfirmed solid-to-solid phase transition prior to the main melting, have been reported in numerous papers [11,12,13,18,19,20,21,23,26]. However, no direct structural identifications of these transitions have been reported, although Jeffrey had proposed that such transitions might be associated with the formation of kinks or with the onset of conformational disorder in the alkyl chains [12]. An understanding of the structural phase transition is essential to determinations of the structural properties, since many physical properties originate from the large fluctuations that accompany phase transitions.

Recently, an exclusive example from among the vast repertoire of crystal structures has been claimed for octyl *β*-D-galactoside (*β*-OGal) hemihydrate, an analogue compound of *β*-OGlu. The crystal structure and phase behavior have been studied effectively by grazing-incidence, wide-angle, X-ray-diffraction (GI-WAXD) analysis [36,37]. By employing GI-WAXD analysis with a two-dimensional detector (2D-GI-WAXD) and using a highly aligned, polycrystalline, sub-μm-thick, glycolipid film assembled on a substrate, the crystal structure of *β*-OGal hemihydrate was shown to have an intermediated ribbon-type crystal structure; this could not have been determined using single-crystal structural analysis. The analysis further exploited the second-order-like phase transition behavior in the anhydrous crystal state with the variation of temperature. Thus, this analytical methodology has effectively increased fundamental understandings of the structural phase transitions of mono-tailed glycoside crystals.

Here, we have applied this methodology to analyze the crystal structure and crystal phase-transition behavior of *β*-OGlu. This molecule formed two types of aligned crystals, which greatly increased the difficulty of the analysis. Fortunately, we have been able to use a thermal treatment to prepare an adequate film for analysis. As a result, the 2D-GI-WAXD analysis has provided us with structural information about the *β*-OGlu crystal. The validity of the identification is supported by analyzing a crystal of n-heptyl *β*-D-glucoside (*β*-HGlu), which we studied for comparison. In addition, the structural properties of the previously unconfirmed solid-to-solid phase transition are also clearly explained by using the combination of the perpendicularly aligned film, out-of-plane X-ray analysis, and 2D-GI-WAXD analysis. In the end, more than 30 years after this was claimed in the pioneering reports by Dorset and Rosenbusch [11] and Jeffrey [12], we have confirmed the concepts of the formation of a unique crystal structure, with a solid-solution-like phase persisting over a wide temperature range, and of the formation of a superlattice with an associated latent heat in a single-substance system.

## 2. Materials and Methods

### 2.1. Glycolipid Samples

We purchased samples of n-octyl *β*-D-glucoside (*β*-OGlu) and n-heptyl *β*-D-glucoside (*β*-HGlu) from Wako Pure Chemical Industries, Ltd. (Tokyo, Japan) and used them without further purification. *β*-OGlu or *β*-HGlu was dissolved in 1:2 (*w/w*) of chloroform/methanol solution.

### 2.2. Assembly of Aligned Glycolipid Film Samples

We prepared each μm-thick glycolipid film by spin-coating 15 wt.% solutions of *β*-OGlu or *β*-HGlu at 4,000 rpm for 50 s onto 2 × 2 cm^2^ Si (100) substrates (Electronics and Materials Corp., Hyogo, Japan). We crystallized each glucoside film in a sealed container at room temperature. Both *β*-OGlu and *β*-HGlu formed well-aligned crystalline films after annealing the spun films at room temperature for 24 h. Each out-of-plane X-ray diffraction (XRD) profile shows only lamellar diffraction spacings, demonstrating the formation of highly perpendicular aligned films (Figure S1, Supplementary Materials). In the present studies, we used films of sub-micrometer thicknesses (Figure S2, Supplementary Materials).

### 2.3. Experimental Procedure

We carried XRD measurements [out-of-plane (2*θ*/*ω*) scans or in-plane (2*θ*/*χ*) scans] using a SmartLab multipurpose X-ray diffractometer (Rigaku Corp., Tokyo, Japan) with a temperature-control unit, under reduced pressure (10 Pa) and employing Cu Kα radiation (0.1542 nm, 40 kV, 40 mA). The one-dimensional electron density profiles in the vertical direction were estimated from the XRD profile corrected using the Lorentz-Polarization (LP) factor. The one-dimensional electron density profiles across the bilayer on the relative electron density scale were estimated from

(1) $$\rho \left(x\right)=\frac{2}{d}\sum_{h=1}^{h=h_{max}}F\left(h\right)cos\left\{\frac{2\pi xh}{d}\right\}$$

where *d* is the bilayer length, *F*(*h*) is the structure amplitude and *h* is the diffraction order of the lamellar phase [38].

We performed two-dimensional GI-WAXD measurements at the BL03XU beamline at SPring-8 (Hyogo, Japan). We obtained diffraction patterns in the range from 0° to 18° by using an imaging-plate detector system (R-AXIS IV, Rigaku Corp.) at the synchrotron-radiation wavelength of 0.100 nm. We performed the measurements in a He atmosphere and used the Fit2D program for data analysis. We performed transmittance small-angle X-ray scattering (SAXS) using a NANO-viewer (Rigaku Corp., Tokyo, Japan) instrument with a temperature-control unit in an N_2_ gas atmosphere and employing Cu Kα radiation (0.154 nm, 40 kV, 40 mA). We obtained the diffractions patterns for powder samples on the glass substrate by using a Pilatus 100K (Rigaku Corp., Tokyo, Japan).

## 3. Results and Discussion

### 3.1. Crystal Structure

Dorset and Rosenbusch [11] reported that the *β*-OGlu crystal exhibited more reflections in the low-angle region than the *α*-OGlu crystal; *α*-OGlu crystal showed a single lamellar repeat. Actually, in the powder X-ray diffraction (PXRD) profiles obtained for this study, we observed several diffraction peaks at lower diffraction angles not only for *β*-OGlu but also for *β*-HGlu (Figure S3, Supplementary Materials). These results evidently exclude the typical glycoside tendency, which obeys *a* >> *c* > *b*. Hence, we considered two possibilities: (i) If *β*-OGlu forms a crystal structure possessing the commonly reported lattice-constant relation *a* (≈ bilayer thickness) >> *c* > *b*, then there are at least two kinds of crystals. (ii) If *β*-OGlu forms an unusual crystal possessing the specific lattice-constant condition *a* ≈ *c* ≈ bilayer thickness, as recently reported for the *β*-OGal hemihydrate crystal [36,37], then a unique single-crystal-type structure must exist. A PXRD profile of a modulated structure of a biomembrane composite produces several diffraction patterns for a single LC arrangement [39,40]. To determine which scenario is valid, we applied 2D-GI-WAXD analysis using the perpendicularly aligned sub-micron-thickness films. Characterisation of the prepared film is described in the Materials and methods section above and in Figures S1 and S2, Supplementary Materials.

**Figure 1:**
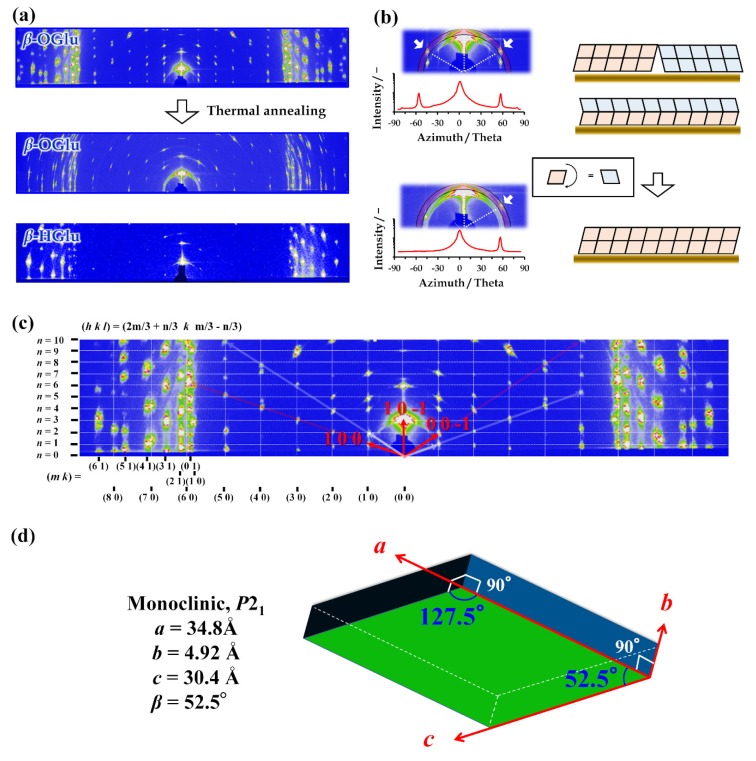
Two-dimensional detector (2D-GI-WAXD) analysis of aligned *β*-OGlu and *β*-HGlu crystalline films. (**a**) 2D-GI-WAXD profiles of (top) film prepared by spin-coating the chloroform/methanol mixed solution (1:2 volume ratio), (middle) the thermally annealed film for *β*-OGlu, and (bottom) the *β*-HGlu as-spun film. (**b**) Representative enlargements of the 2D-GI-WAXD profiles (left) and schematic illustrations of twin-crystal formations (right). (**c**) Assignment of the *β*-OGlu profile. The red and white arrows discriminate the basic vectors of aligning crystals in the twining state. (**d**) Schematic illustration of the crystal parameters.

Figure 1 summarizes the results of the 2D-GI-WAXD analyzes of the *β*-OGlu crystal films at 30 °C. The profile for the as-prepared *β*-OGlu film clearly shows numerous organized reflection spots, even without rotating the sample (Figure 1a, top), demonstrating the formation of a polycrystalline structure aligned to the substrate. However, it was difficult to determine the crystal-lattice parameters from the profile. Fortunately, we found that when the film was heated to just below *T*_m_ and subsequently cooled to 30 °C, the profile had greatly changed. We obtained an unsymmetrical profile along the 0° azimuth for the thermally treated film (Figure 1a, middle), while we obtained symmetrical reflection patterns for the profile of the as-prepared film (Figure 1a,b, top). These results support the assumption that the symmetrical profile can be explained if the *β*-OGlu forms two differently aligned crystals on the substrate (Figure 1b, above). This makes *β*-OGlu single crystals very difficult to prepare and explains the absence of any published report solving the structure. On the other hand, for *β*-HGlu, the 2D-GI-WAXD profile we obtained from the initial film is unsymmetrical (Figure 1a,b bottom). The unsymmetrical profile helped greatly in assigning the (1, 0, 0) and (0, 0, −1) directions, as shown in Figure 1c. All the reflections in the wide-angle diffraction ranges could be well indexed as (2m/3 + n/3, k, m/3 - n/3), with m, k and n as the integers. From these results, we obtained the crystal-lattice parameters *a* = 34.8 Å, *b* = 4.92 Å, *c* =30.4 Å and *β* = 52.5° (Figure 1d). Considering the extinction law, we assumed the space group to be *P*2_1_. For the *β*-HGlu crystal, we also determined the crystal-lattice parameters to be *a* = 33.1 Å, *b* = 4.92 Å, *c* = 28.7 Å and *β* = 55.5°, and we again assumed the space group to be *P*2_1_. The diffraction angles at the locations y = 0, 1, 2 and 3 were very similar for both *β*-OGlu and *β*-HGlu in the in-plane directions relative to the out-of-plane axis (Figure S4, Supplementary Materials). This strongly suggests that the crystal structures must be basically similar, with the only variation along the out-of-plane axis being the molecular-length difference between *β*-OGlu and *β*-HGlu. This difference is 1.93Å, and it corresponds well to the perpendicular alignments of the alkyl chain relative to the substrate, with a maximal tilt of 40~41° to the normal to the bilayer [34]. The one-dimensional electron density profile obtained from the corrected XRD for the LP factor supported the finding. At the least, the existence of bilayer structure in the perpendicularly aligning was recognized for both crystal and LC phase (Figure S5, Supplementary Materials). Moreover, no distinct difference in the electron density profile for the head groups between *β*-OGlu and *β*-HGlu crystals were observed, also supporting the findings that these crystal structures were very similar without the length of alkyl chain.

On the other hand, considering the model proposed for LC structures [40,41] and the above assumption, we assumed the crystalline film to have a modulated-bilayer, in which the lamellar structure is aligned perpendicular to the substrate. To the best of our knowledge, such a modulated bilayer crystal has never previously been reported for glycolipid crystals. But, similar to the *β*-OGal hemihydrate crystal, we expect a Z value larger than 12 for *β*-OGlu. This means that there are several *β*-OGlu molecules playing different roles or being located in different frustrating situations, even in a pure-substance crystal. This demonstrates that the crystal forms via versatile cooperative self-assembly, which must be a highly specific behavior for glycolipid crystals. As reported for lipid bilayers, an intermediate phase—like a ripple phase—must form via the driving force that causes lipid molecules with large head groups to splay [42,43]. Thus, *β*-OGlu must be intrinsically equipped with such a structure, with a large splayed head group, and must form an intermediate molecular assembly, even in the crystalline state.

### 3.2. Crystal Phase Transition Behavior

**Figure 2:**
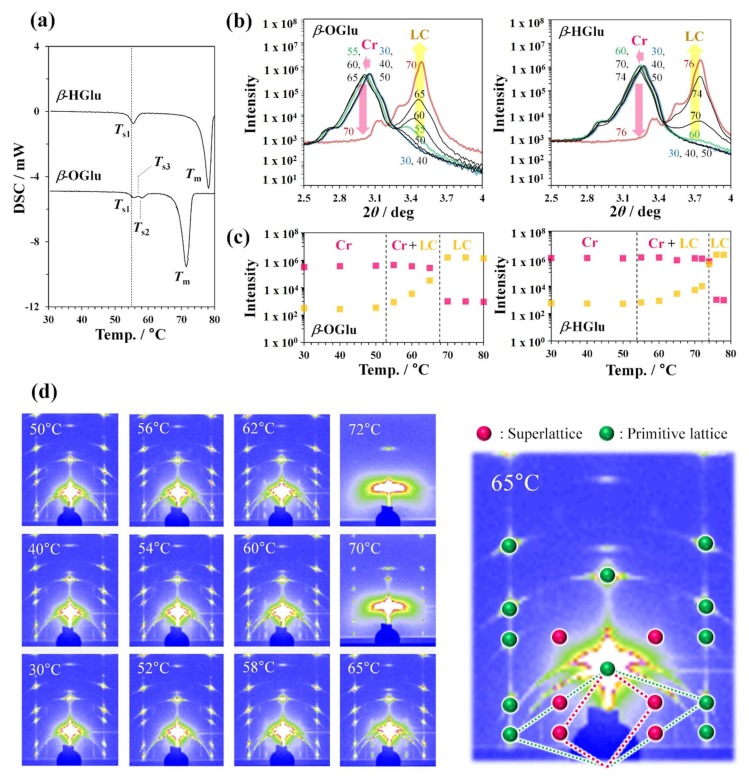
The solid-to-solid phase transitions. (**a**) DSC thermograms of *β*-OGlu and *β*-HGlu. The temperatures corresponding to the solid-to-solid phase transitions are termed *T*_s_ in this article. In the thermogram for *β*-OGlu, two endothermic peaks (*T*_s1_ and *T*_s2_) or one endothermic peak (*T*_s1_) and one exothermic peak (*T*_s3_) exist. (**b**) Temperature-dependent PXRD profiles at low diffraction angles and (**c**) the corresponding peak intensities. (**d**) 2D-GI-WAXD analysis of crystalline *β*-OGlu films. 2D-GI-WAXD profiles of (left) films under temperature-controlled conditions and (right) an extended profile obtained at 65 °C.

Figure 2a shows the thermograms of *β*-OGlu and *β*-HGlu obtained by a differential-scanning-calorimeter (DSC) measurement, similar to in a previous study [26]. In the heating thermograms, we observed the main melting to occur around 68–74 °C and 76–80 °C for *β*-OGlu and *β*-HGlu, respectively. In addition, between 50 and 60 °C, we recognized several thermal peaks in both thermograms, which are attributed to solid-to-solid phase transitions [11,12,13,18,19,20,21,23,26]. The temperature corresponding to a solid-to-solid phase transition is termed *T*_s_ in this article.

Since the PXRD profiles obtained from the powder samples did not afford meaningful results (Figure S6, Supporting Information), in order to obtain better information, we carried out temperature-dependent X-ray analyses of the perpendicularly aligned assembled samples. In the temperature-dependent out-of-plane XRD profiles for those aligned crystalline films, we ascribed the diffraction peaks around 3.5° for *β*-OGlu and 3.7° for *β*-HGlu to the formation of an LC phase (Figure 2b). Surprisingly, the partition of the LC phases seems to start increasing around 50 °C around *T*_s_, even though the values of *T*_m_ were around 68 °C and 74 °C, respectively (Figure 2c). Thus, in those systems, a binary phase consisting of an LC and a crystalline phase formed over a wide temperature range, and the occurrence of an endothermic peak signifies a phase transition between a single-crystal-type phase and a solid-solution-like phase consisting of a crystalline and an LC phase. The occurrence of the LC phase must result from the conformation transformation of the alkyl-chain domains, as reported for n-alkane-chain lipids [44,45] and assumed by Jeffrey [10].

On the other hand, as shown in Figure 2a, we observed several thermal phenomena as solid-to-solid phase transitions for the *β*-OGlu crystal, whereas only one endothermic peak was observed for the *β*-HGlu crystal. This difference cannot be explained by the PXRD analysis. To clarify the difference, we therefore performed temperature-dependent 2D-GI-WAXD analyses.

Figure 2d shows the temperature-dependence of the GI-WAXD profiles for *β*-OGlu. As the temperature increased above *T*_s2_, we identified the reflections from the superlattice structure consisting of the vectors (1, 0, −1/2) and (0, 0, −1/2) (Figure 2d). Note that superlattice formation as well as partial melting behavior was also recognized for the powder sample in the SAXS analysis (Figure S7, Supporting Information), demonstrating that this is not a film-specific phenomenon. Superlattice formation for the *β*-OGlu crystal was accompanied by changes in the longer-range orderings. The rearrangement and/or thermal expansion of the alkyl chains must cause the head groups to reorient to reduce the order parameter developed by the *β*-OGlu aggregates. Such a large distinct change in the head geometry must be case-specific to the glycolipid study.

## 4. Conclusions

Crystal-structure analyses of the glycolipids were started in part to serve as relevant model systems for the complex structural transitions that occur in the functioning of the glycolipids found in cell membranes (Jeffrey, 1986). However, the crystal structure obtained was always assumed to consist of a simple lamellar assembly instead of being derived from a complicated cooperative molecular assembly, such as an intermediate structure that is often observed in natural lipid LC systems. In contrast to such previous reports, we strongly claim that such structures can be observed in the crystalline state. However, it has been difficult to perform single-crystal structural analyses, because crystals of adequate size could not be readily obtained. In the case of *β*-OGlu, the formation of twin crystals must impede this analyis. However, the introduction of 2D-GI-WAXD analysis and the use of perpendicularly aligned films has revealed the previously unknown facts that *β*-OGlu forms a modulated crystal and a superlattice.

The crystal-structure information obtained in this paper affords an understanding of the fundamental structural phase transitions of glycolipids, which somewhat overturns the previous general consensus concerning these compounds that was developed without the knowledge of the *β*-OGlu crystal. We find that the glycolipid crystal forms a complicated crystal state with a modulation, like the intermediate ripple state observed for natural lipids in the LC phase, whereas a simple lamellar packing was always proposed previously for the glycolipid crystal structure revealed by single-crystal structural analysis.

We consider partial melting as one sign of a solid-to-solid phase transition, which starts to form solid-solution-like behavior consisting of a crystalline and an LC phase. In addition, the rearrangement of the two-dimensional sugar structure also contributes to the solid-to-solid phase transition, where the superlattice formation can be detected. These complicated behaviors occur in a pure substance; therefore, each individual *β*-OGlu molecule plays a versatile role in a single-component phase in the crystal and its melting process.

## Supplementary Materials

The following materials are available online at https://www.mdpi.com/2073-4360/12/3/671/s1, Figure S1: Out-of-plane XRD profiles of film samples at room temperature, Figure S2: XRR profiles of film samples at room temperature, Figure S3: XRD analysis of powder samples at room temperature, Figure S4: Comparison of out-of-plane and in-plane XRD profiles for film samples, Figure S5: One-dimensional electron density profile in the vertical direction to the Si substrate, Figure S6: XRD analysis of powder samples at different temperatures, Figure S7: Temperature-dependent 2D-SAXS profiles of *β*-OGlu powder samples at different temperatures.
