# Supplementary material for: Unveiling the Uniqueness of Crystal Structure and Crystalline Phase Behavior of Anhydrous Octyl β-D-Glucoside Using Aligned Assembly on a Surface
Source: Polymers (Basel). 2020 Mar 17;12(3):671. doi: 10.3390/polym12030671 (PMC7183315; doi:10.3390/polym12030671)
Supplement: Supplementary file 1 [file polymers-12-00671-s001.pdf]

SUPPLEMENTARY MATERIAL

Unveiling the uniqueness of crystal structure and crystalline phase behaviours of anhydrous octyl *β*-D-glucoside using aligned assembly on a surface

Shigesaburo Ogawa[1], Isao Takahashi[2]

[1] *Department of Materials and Life Science, Faculty of Science and Technology, Seikei University, 3-3-1 Kichijojikitamachi, Musashino-shi, Tokyo, 180-8633, Japan*

[2] *Department of Physics, School of Science and Technology, Kwansei Gakuin University, Sanda 669-1337, Japan.*

—Contents of the Supplementary Material—

I) X-ray diffraction analysis of film sample at room temperature

II) X-ray diffraction analysis of powder sample at room temperature

III) Out-of-plane and in-plane X-ray analyses of film sample

IV) Estimation of electron density profile in vertical direction to Si substrate

V) X-ray diffraction analysis of powder sample under temperature-controlled conditions

IV) Small-angle X-ray scattering analysis of powder sample

I) X-ray diffraction analysis of film sample at room temperature

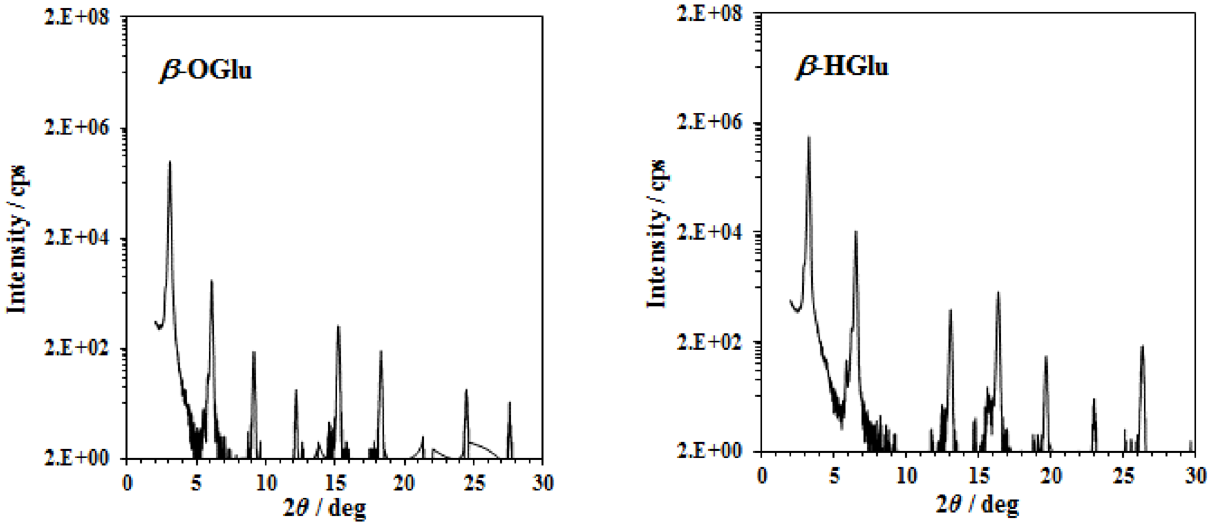

Figure S1. Out-of-plane XRD profiles of *β*-OGlu and *β*-HGlu films prepared from 15 wt% solutions on Si (100) substrates.

Here, the solution concentration used for spin-coating was 15 wt%. We determined the film thickness to be some seven hundred nm by considering the relationship between film thickness and the concentration used for spin-coating. For instance, we determined the film thicknesses prepared from 1.5 wt% solutions from X-ray-reflection (XRR) analysis to be 71.6 nm for *β*-OGlu and 75.0 nm for *β*-HGlu (Figure S2). Therefore, by using a ten-times-larger concentration, we assume that about ten times this thickness will be obtained. We carried out 2D-WAXD analyses and in-plane XRD analyses of these samples,

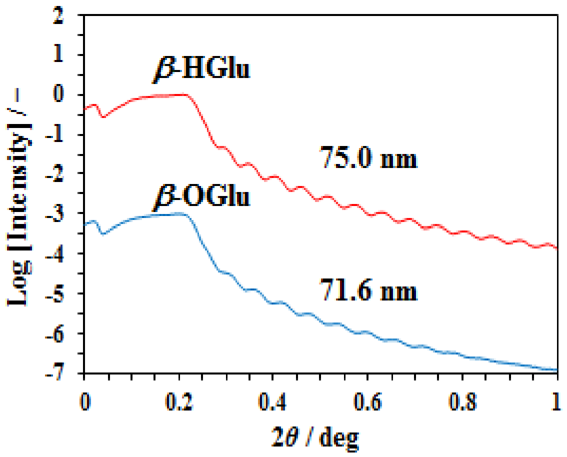

Figure S2. XRR profiles of *β*-OGlu and *β*-HGlu films prepared from 1.5 wt% solutions on Si (100) substrates.

II) X-ray diffraction analysis of powder sample at room temperature

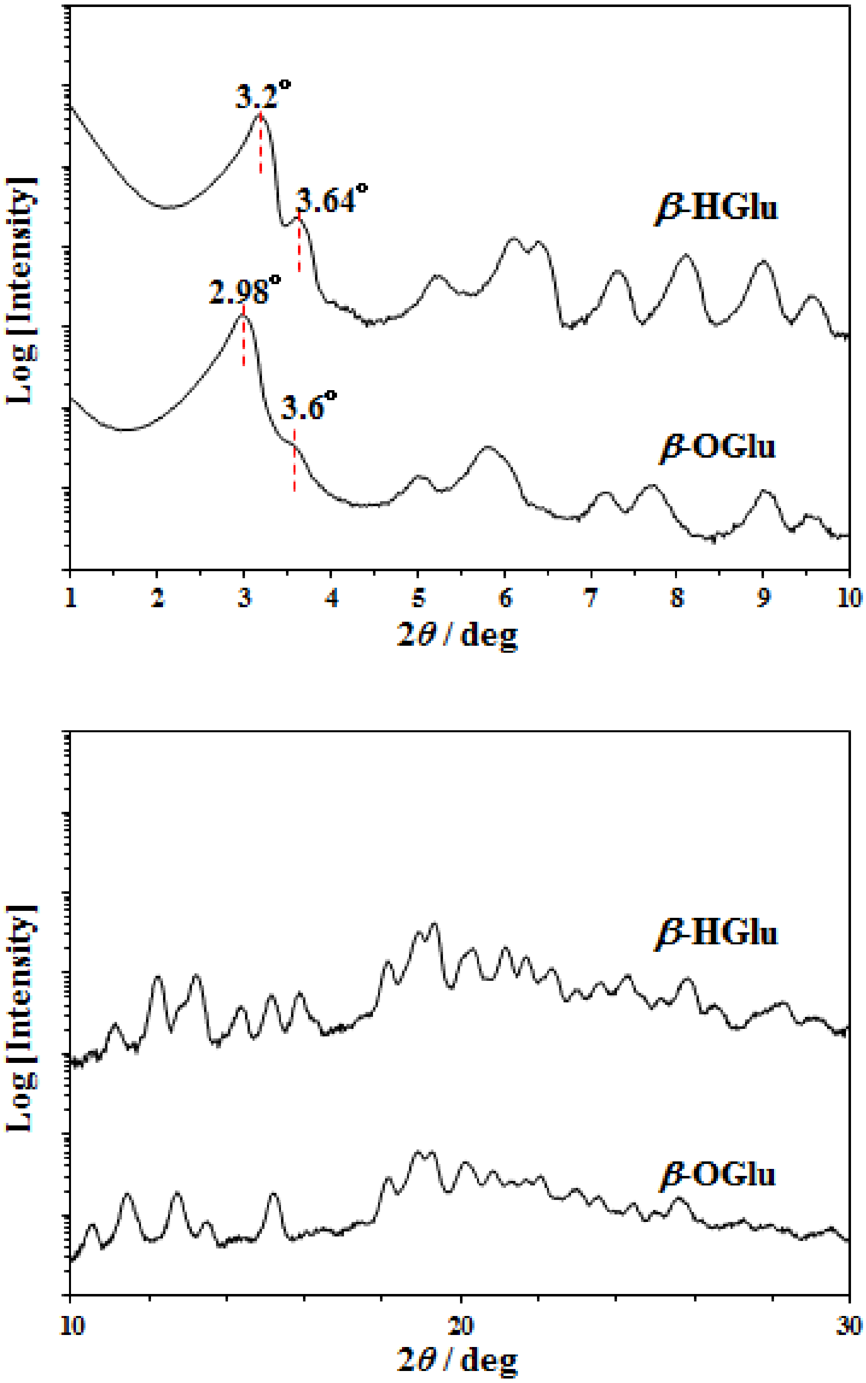

Figure S3. XRD analysis of *β*-HGlu and *β*-OGlu powders at room temperature. The clear diffraction peaks at wide angles show that the samples are in crystalline states. The several diffraction peaks at lower angles show that these crystals are in mixed states or specific.

III) Out-of-plane and in-plane X-ray analyses of film sample

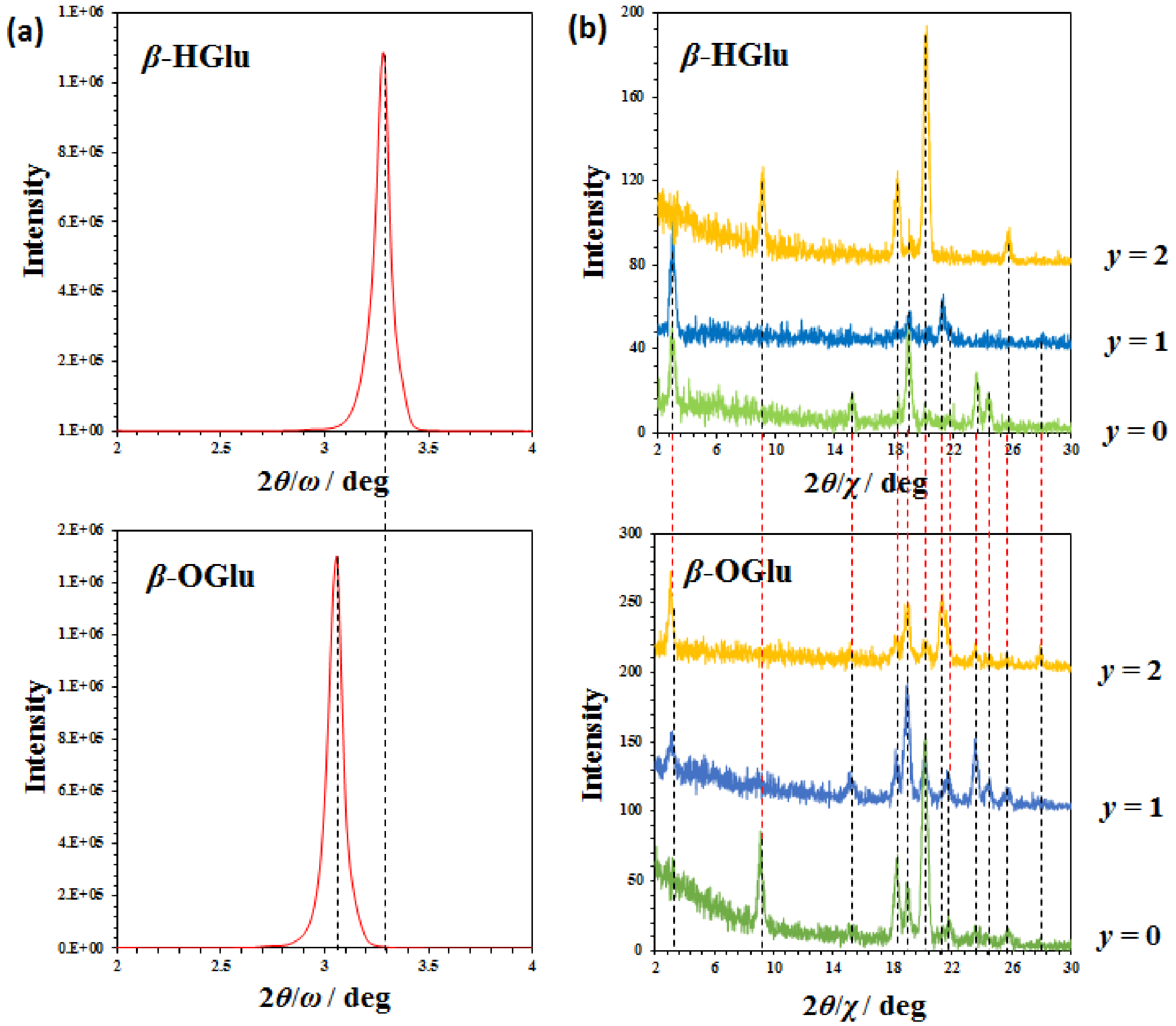

Figure S4. Comparison of (a) out-of-plane and (b) in-plane XRD profiles for *β*-HGlu and *β*-OGlu films.

IV) Estimation of electron density profile in vertical direction to Si substrate

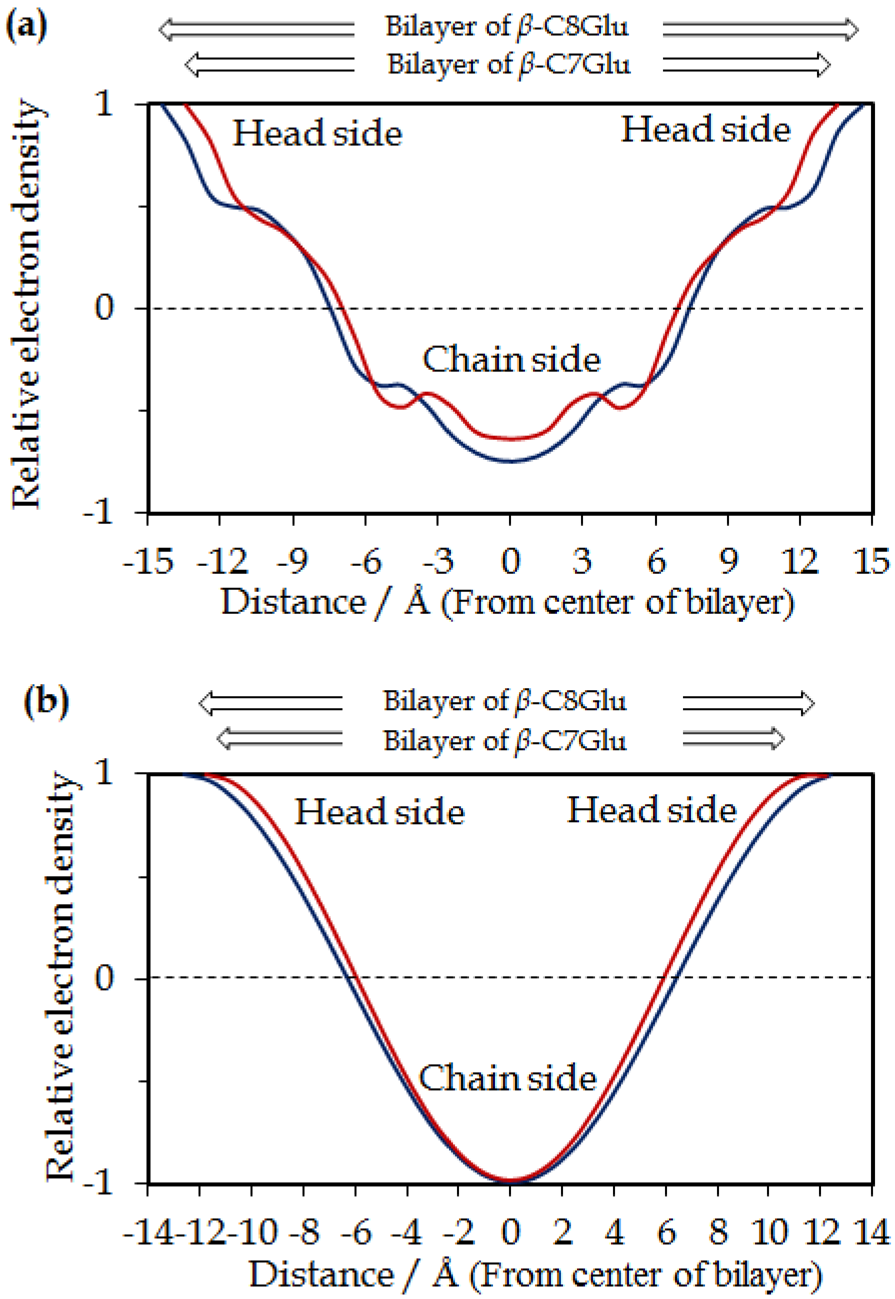

Figure S5. One-dimensional electron density profile in the vertical direction to the Si substrate. (a) Crystal states at 30°C and (c) LC states at 80°C of *β*-HGlu (red) and *β*-OGlu (blue), respectively. Due to the large thermal fluctuation persisting the bilayer structure in the LC phase, the electron densities for the LC phases were estimated as average waves.

V) X-ray diffraction analysis of powder sample under temperature-controlled conditions

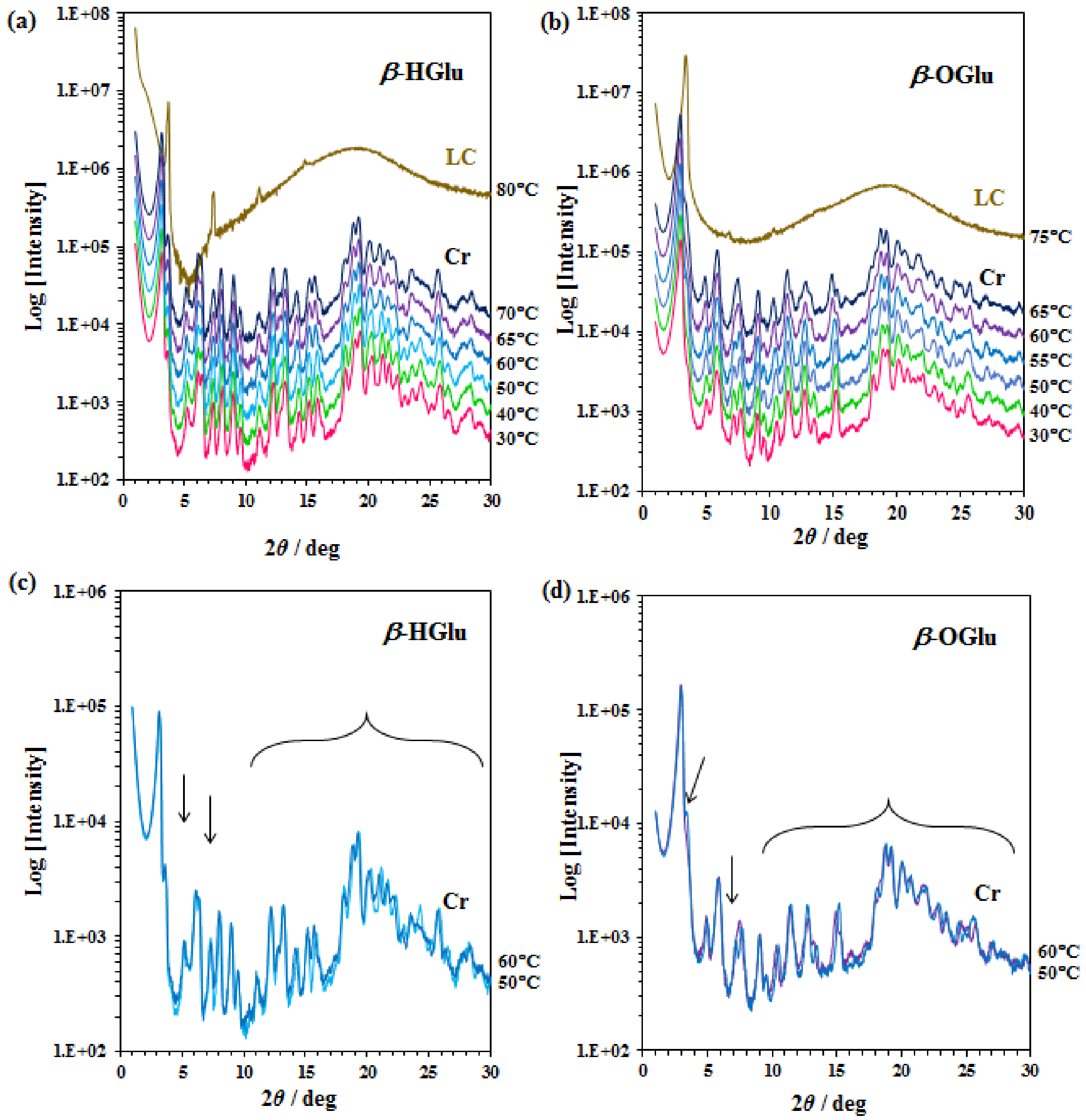

Figure S6. XRD analysis of (a, c) β-HGlu and (b, d) β-OGlu powder samples at different temperatures. Slight differences (indicated by arrows) occur in the two profiles obtained above and below the temperature where the endothermic peaks attributed to the solid-to-solid phase transition during heating appears in the DSC thermogram, but detailed information was difficult to extract from these results.

VI) Small-angle X-ray scattering analysis of powder sample

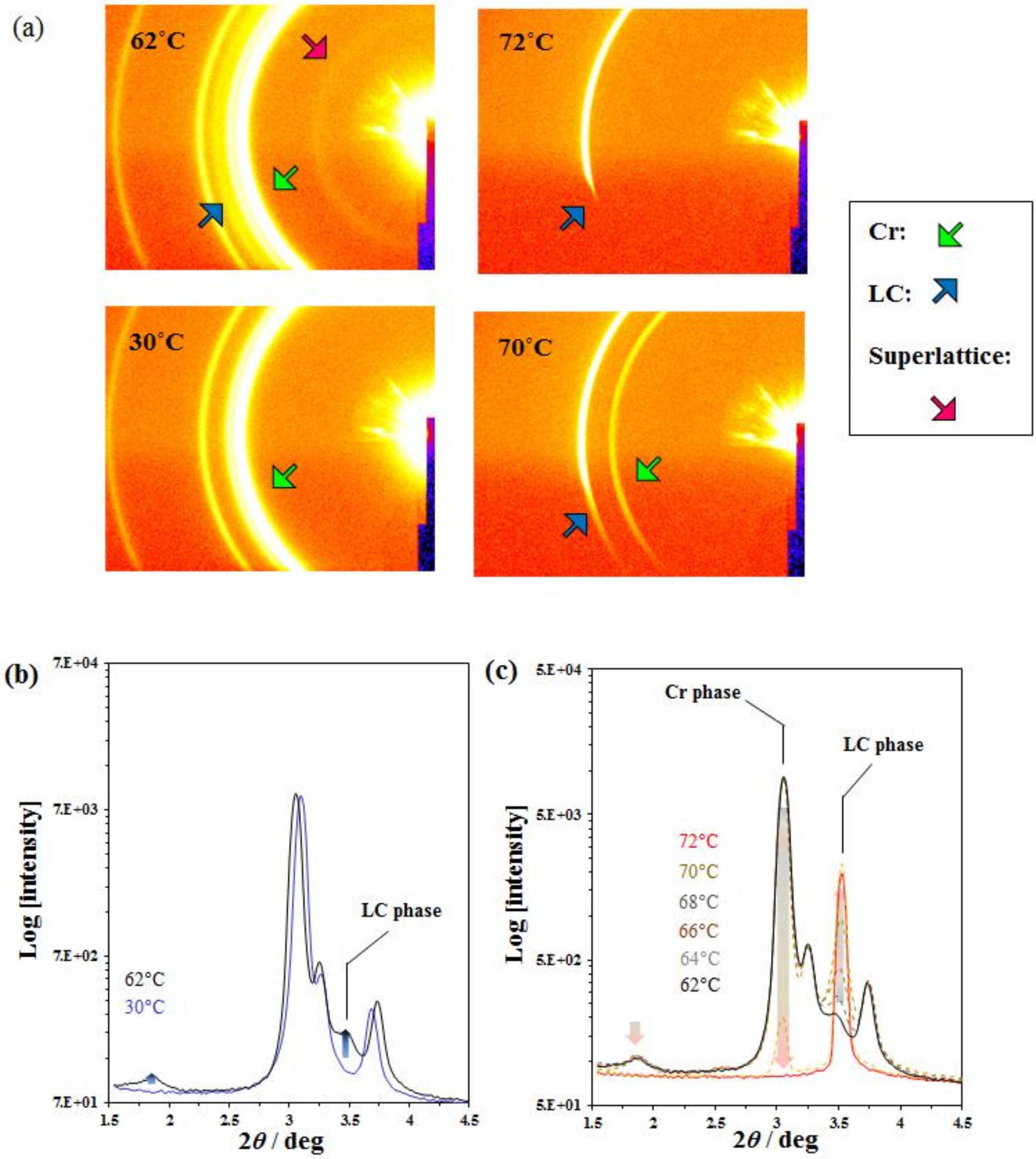

Figure S7. Temperature-dependent 2D-SAXS profiles of β-OGlu powder samples at different temperatures. (a) 1D-SAXS profiles at different temperatures (b) lower than 62°C and (c) higher than 62°C.
